# Understanding the Role of Staff Nurses in Hypertension Management in Primary Care Facilities in India: A Time-Motion Study

**DOI:** 10.5888/pcd20.220232

**Published:** 2023-05-18

**Authors:** Ashish Krishna, Sharan Murali, Andrew E. Moran, Ashish Saxena, Sandeep Singh Gill, Dagmara Hering, Prabhdeep Kaur

**Affiliations:** 1 Indian Council of Medical Research — National Institute of Epidemiology, Chennai, India; 2 Resolve to Save Lives, New Delhi, India; 3 Resolve to Save Lives, New York, New York; 4 Columbia University Irving Medical Center, New York, New York; 5 Directorate of Health Services, Government of Madhya Pradesh, Bhopal, India; 6 Department of Health and Family Welfare Punjab, Chandigarh, Punjab, India; 7 Department of Hypertension and Diabetology, Medical University of Gdansk, Gdansk, Poland

## Abstract

**Introduction:**

India is facing a shortage of staff nurses; thus, a better understanding of nurses’ workloads is essential for improving and implementing noncommunicable disease (NCD) control strategies. We estimated the proportion of time spent by staff nurses on hypertension and other NCD activities in primary care facilities in 2 states in India.

**Methods:**

We conducted a cross-sectional study in 6 purposively selected primary care facilities in Punjab and Madhya Pradesh during July through September 2021. We used a standardized stopwatch to collect data for time spent on direct hypertension activities (measuring blood pressure, counseling, recording blood pressure measurement, and other NCD-related activities), indirect hypertension activities (data management, patient follow-up calls), and non-NCD activities. We used the Mann-Whitney *U* test to compare the median time spent on activities between facilities using paper-based records and the Simple mobile device–based app (open-source software).

**Results:**

Six staff nurses were observed for 213 person-hours. Nurses spent 111 person-hours (52%; 95% CI, 45%–59%) on direct hypertension activities and 30 person-hours (14%; 95% CI, 10%–19%) on indirect hypertension activities. The time spent on blood pressure measurement (34 minutes) and documentation (35 minutes) was the maximum time on any given day. Facilities that used paper records spent more median time (39 [IQR, 26–62] minutes) for indirect hypertension activities than those using the Simple app (15 [IQR, 11–19] minutes; *P* < .001).

**Conclusion:**

Our study found that hypertension activities required more than half of nurses’ time in India’s primary care facilities. Digital systems can help to reduce the time spent on indirect hypertension activities.

**Summary**

**What is already known on this topic?**

Nurses are responsible for multiple tasks in primary care facilities in India. However, understanding of the type of work and the time spent on various activities nurses perform for hypertension management is limited.

**What is added by this report?**

Nurses spent two-thirds of their time on hypertension activities in the 6 primary care noncommunicable disease clinics in India. Nurses in clinics using paper-based records took more time than did nurses in clinics using digital health records.

**What are the implications for public health practice?**

Primary care facilities require substantial time commitment by nurses to improve hypertension management in India. Using digital health records increases nurses’ productivity and should be promoted to improve patient care.

## Introduction

Cardiovascular diseases (CVD) are the leading global cause of increased illness and death (1). Hypertension is the leading risk factor for CVD (2). The prevalence of hypertension in adults aged 18 to 69 was 29% in India in 2017 through 2018 (3). Among patients with hypertension, only 12% had their blood pressure controlled, highlighting an urgent need to strengthen hypertension control services in India (3). Health care systems worldwide use a combination of interventions to prevent hypertension and improve its control, thus preventing the development of associated CVD (4,5). One intervention is to have an organized system of regular follow-up and medication review by health care professionals other than doctors, such as nurses and pharmacists (4). A meta-analysis of 31 interventional studies in low-income and middle-income countries showed that task sharing with nurses decreased blood pressure by 5.34 mm Hg (6).

In India, hypertension screening and treatment are part of the National Programme for Prevention and Control of Cancer, Diabetes, Cardiovascular Diseases, and Stroke. Nurses are assigned to conduct various activities under this program. Nurses conduct opportunistic screening for adults aged 30 years or older who visit noncommunicable disease (NCD) clinics and refer patients to a doctor if their systolic blood pressure is 140 mm Hg or higher and/or their diastolic blood pressure is 90 mm Hg or higher. Nurses also measure blood pressure, document the visit on the treatment card (digital or paper), and counsel patients on treatment of hypertension (7). However, there is a shortage of nurses, as shown by the nurse-to-population ratio of 1.7:1,000 in India. The World Health Organization recommends a nurse-to-population ratio of 3:1,000 in each country to achieve universal health coverage (8). Thus, nurses have to multitask because of the unavailability of a trained workforce in the NCD clinics, leading to compromises in patient care delivery (9).

Primary care facilities use 2 different types of patient monitoring systems in India. The first is the conventional paper-based system, where the nurses record the patient data on a printed treatment card. The second method is a digital system where the nurses record the patient data on a mobile device–based application synced with a central server. The digital system enables the nurse to retrieve the data for their patients from the server. The Simple app (open-source software) is a digital technology used for patient tracking and monitoring in selected states in India (10,11).

Considering the urgent need to scale hypertension treatment with the available nurses, a better understanding of nurse workload is essential for improving and implementing strategies. Evidence is limited about the time distribution for nurses’ activities in NCD clinics in India. Time and motion studies calculate efficiency, simplify time-consuming processes, and eliminate repetitive tasks (12). Researchers worldwide have used this method to measure time spent on various activities to understand the workflow of pharmacists, physicians, and nursing staff in hospitals and clinics in multiple settings (13).

Therefore, we designed and conducted this time and motion study to estimate the proportion of time spent by the staff nurses on hypertension and other NCD activities in primary care facilities in 2 states in India. We also compared the time spent on hypertension and other NCD activities in the facilities using paper-based documentation systems with the facilities using the Simple app-based digital system.

## Methods

### Study design and setting

We conducted a cross-sectional study in 6 purposively selected public sector NCD clinics in the primary care facilities in Madhya Pradesh and Punjab during July 2021 through September 2021. We selected 3 facilities in each state, considering feasibility and logistics. NCD clinics deliver services for diagnosis, treatment, and follow-up of hypertension and diabetes, operate all weekdays during the forenoon hours (8:30 am to 2:00 pm), and have approximately 50 to 200 patients daily. All 6 clinics participate in the India Hypertension Control Initiative (IHCI), which aims to strengthen the management of hypertension in primary care settings in select districts in India. The nurses in these clinics are trained and follow the IHCI screening, treatment, and follow-up guidelines (14). Individuals aged 30 years or older with hypertension diagnosed by a physician are registered into the IHCI program by the individual facilities. Nearly three-fifths of the population were aged 50 to 60 years and were women (15).

The 3 clinics in Madhya Pradesh use a paper-based documentation system for registration, follow-up, and patient monitoring. The staff nurses fill out treatment cards for each enrolled patient. Nurses store the treatment cards at the health care facility and retrieve them during each follow-up visit. Treatment cards are the source of NCD data, which are compiled to prepare reports for sending to the district NCD cell.

The 3 clinics in Punjab use the digital mobile-based app Simple to monitor their patients. Upon registration, each patient receives a BP Passport, which carries a quick response (QR) code unique to the patient (10). The QR code is scanned during every visit to load the patient data (blood pressure, capillary blood glucose, and medications) onto the Simple app. This app also works offline and syncs its data to the server later when the internet is available. The Simple dashboard can quickly create data downloads and reports for the district NCD cell.

### Study population

Each selected health facility had 1 dedicated paramedical staff nurse or an available staff nurse designated for the NCD clinic. This NCD nurse, who was in charge of direct and indirect patient care, was observed as a part of this study.

### Sample size and assumptions

We assumed that staff nurses spend approximately 50% of their time in the NCD clinic on hypertension activities. Using this assumption, we calculated the required sample size for the primary objective with a 95% CI and an absolute precision of 10% as 97 hours. The duty time for each nurse in the NCD clinic is approximately 4 hours per day. Hence we observed each of the 6 nurses for a minimum of 4 days to achieve the sample size. We used OpenEpi version 3.1 (open source software) to calculate the sample size (16).

### Data collection

We observed each facility for at least 7 days, considering the daily variability in the number of patients and the nursing workload during the week. We trained 2 full-time research assistants to collect data using a paper-based tally sheet in all 6 clinics. They collected data for each nurse, including age, sex, years of work experience treating patients with NCDs, and details of training for hypertension management. The research assistants observed the activities of the staff nurses during the clinic working hours and recorded the time taken for each category of activity by using Google Clock on the Android mobile telephones of the data collectors to record the time. The study team observed the various activities performed by the staff nurse and categorized activities on the basis of whether the activities were directly linked to patient care or were related to other programmatic needs.

We operationally defined blood pressure measurement, counseling, recording blood pressure measurement, and other NCD-related activities (electrocardiogram, capillary blood glucose measurement, drug dispensing, and follow-up of patients with diabetes) as direct hypertension activities. Indirect hypertension activities were data management (updating patient records) and follow-up calls. We also measured activities unrelated to NCDs, including delivering care to antenatal mothers, nonspecific administrative duties, and idle time (no work) (Table 1). We did not collect any patient-related data from the facilities.

**Table 1:** Operational Definitions of Categories of Key Activities Performed by Staff Nurses in NCD clinics of Selected Primary Care Facilities of Madhya Pradesh and Punjab, 2021

Category	Activity	Description
Direct hypertension activities	BP measurement	Time to place the BP cuff on the patient’s arm, measure the BP, and remove the cuff.
	Counseling	Time taken to give instructions to patients regarding medication use and lifestyle modification.
	Recording BP measurements	Time taken to record the measured BP to the registers or in the Simple^a^ app.
	Other NCD-related activities	Time taken during the following activities was collated under this heading, because these tasks were not performed for all the patients: • **Drug dispensing:** Time taken dispensing drugs per the protocol and documenting the drug administration information on a treatment card or in the Simple app.^a^ • **ECG:** Time taken measuring the ECG of the patient, starting from attaching the ECG leads to removing them. • **Blood glucose measurement**: Time taken for pricking the patient’s finger, loading the blood spot onto the glucometer, and retrieving the result. • **Follow-up of patients with diabetes.**
Indirect hypertension activities	Patients record and data management	• Time spent organizing, retrieving, and updating patient information (BP, ECG, and blood glucose) and documenting it on a treatment card or in the Simple app.^a^ • Time spent tallying the patient details and preparing reports for the district NCD cell.
	Making follow-up calls to patient	Time spent accessing the telephone number, calling the patient, and planning the next follow-up call.
Non-NCD activities	Other activities	Time spent having lunch, delivering care to antenatal mothers, and any nonspecific administrative duties.
	No work	Total time when the staff nurses were idle without any specific work.

Abbreviations: BP, blood pressure; ECG, electrocardiogram; NCD, noncommunicable diseases.

a Open source software (10).

### Statistical analysis

We entered the data from the paper tally sheets into Excel (Microsoft). We calculated the mean time a staff nurse spent per day in the clinic by dividing the total time observed for a specific nurse by the number of days observed for each facility. We analyzed the data separately for primary care facilities in Madhya Pradesh and Punjab to calculate the mean (SD) time spent by a staff nurse per day in the clinic.

We present the sociodemographic characteristics of nursing staff using descriptive statistics. We calculated the total time spent on defined activities as the median (IQR) time spent per day per nurse for each activity. We calculated the median (IQR) time spent per day on direct, indirect, and non-NCD activities. Total time spent for each category was divided by the total observed time to estimate the proportion of time taken with its 95% CI.

We separately summarized the median (IQR) time for direct hypertension, indirect hypertension, and non-NCD activities for facilities using paper-based systems and facilities using Simple. We compared the median time taken across the 2 types of facilities by using the Mann-Whitney *U* test. A *P* value less than .05 was considered significant. We used Stata SE (version 17) software (StataCorp LLC) for statistical analysis.

### Ethical considerations

We obtained approval from the Institutional Ethics Committee of the Indian Council of Medical Research — National Institute of Epidemiology. The research assistants obtained written informed consent from staff nurses after explaining the study procedure. No patient data were collected or analyzed in this study. Data were de-identified at the collection level and stored under the confidentiality of the corresponding author.

## Results

Among the 6 observed primary care facilities, 3 clinics had dedicated NCD staff nurses who performed NCD activities. At the other 3 facilities, the staff nurse also performed non-NCD activities, including general outpatient department management, wound dressing, and administrative duties. The mean time spent per day by staff nurses in each of the 3 clinics was 4 hours 25 minutes (SD, 1 hour 35 minutes) in Madhya Pradesh and 3 hours 40 minutes (SD, 25 minutes) in Punjab (Table 2).

**Table 2:** Characteristics of Selected Noncommunicable Disease Clinics in Selected Primary Care Facilities of Madhya Pradesh and Punjab, 2021 (N = 6)

Facility code	Availability of dedicated staff	Total time observed, min	Total days observed	Mean time spent by a staff nurse per day in the clinic, min	Mean (SD) hours spent by a staff nurse per day in the clinic
Madhya Pradesh using a paper-based system					
Facility 1	Yes	1,923	10	193	4 h 25 min (1 h 35 min)
Facility 2	Yes	3,730	10	373	
Facility 3	No	2,307	10	231	
Punjab using Simple^a^ app					
Facility 1	No	1,525	8	191	3 h 40 min (25 min)
Facility 2	No	1,644	7	235	
Facility 3	Yes	1,638	7	234	

a Open source software (10).

The staff nurses’ mean (SD) age was 37 (8) years, and 5 were women. The mean (SD) years of work experience were 12 (6) years. All 6 nurses had training in screening, treatment protocol, and follow-up management for diabetes and hypertension patients at the clinic. Five facilities had an outpatient load of more than 100 patients per day, while 1 had a load of fewer than 100 patients per day.

### Time spent on hypertension activities

We observed the 6 nurses for 52 days, accounting for 213 person-hours. All 6 nurses spent a total of 111 person-hours (52%; 95% CI, 45%–59%) on direct hypertension activities, 30 person-hours (14%; 95% CI, 10%–19%) on indirect hypertension activities, and 72 person-hours (34%; 95% CI, 28%–41%) on non-NCD activities (Table 3).

**Table 3:** Time Spent by the Staff Nurse on Each Activity and the Proportion of Time Spent on Various Categories of Activities in the NCD Clinics of Selected Primary Care Facilities of Madhya Pradesh and Punjab, 2021

Category of activity	Activity	Total time, min	Time taken per day, min		Proportion of total time, % (95% CI)
			Median	IQR	
**Direct hypertension activities**	Blood pressure measurement	2,106	34	25–48	52 (45–59)
	Counseling	1,703	23	13–36	
	Recording blood pressure measurement	2,289	35	23–57	
	Other NCD-related activities	525	8	3–21	
**Indirect hypertension activities**	Data management (updating patient records)	1,367	37	21–60	14 (10–19)
	Making patient follow-up calls	457	15	12–23	
**Non-NCD activities**	Other activities^a^	1,897	28	16–57	34 (28–41)
	Idle time	2,424	85	54–112	

Abbreviation: NCD, noncommunicable disease.

a Other activities include lunch, personal breaks, antenatal care delivery, and administrative work.

The median time spent directly on hypertension activities per day per staff nurse was 1 hour 45 minutes (IQR, 1 hour 18 minutes to 2 hours 45 minutes). Nurses spent 24 minutes (IQR, 15 to 44 minutes) per day for indirect hypertension activities and 1 hour 12 minutes (IQR, 50 minutes to 1 hour 56 minutes) per day for non-NCD activities.

Among the direct hypertension activities per day, measurement (34 [IQR, 25–48] minutes) and documentation (35 [IQR, 23–57] minutes) of blood pressure took the most time, followed by counseling (23 [IQR, 13–36] minutes). The indirect hypertension activity of data management (updating patient records) took 37 minutes daily. The staff nurses spent 28 minutes daily on activities unrelated to the NCD clinic (Table 3).

### Application-based versus paper-based records

The median time taken for direct hypertension activities was comparable in the paper-based (110 [IQR, 71–195] minutes) facilities and the facilities using the Simple app (101 [IQR, 71–124] minutes) (*P* = .22) (Table 4). However, the median time spent on indirect hypertension activities was much longer in the paper-based facilities (39 [IQR, 26–62] minutes) when compared with facilities using the Simple app (15 [IQR, 11–19] minutes); a difference of 24 minutes more on each workday in facilities using a paper-based information system (*P* < .001).

**Table 4:** Comparison of Time Spent by the Staff Nurse on Each Activity Category Using Paper-Based Documentation Versus Simple^a^ App in the Clinics of Selected Primary Care Facilities of Madhya Pradesh and Punjab, 2021

Category of activity	Time taken per day, min				*P* value^b^
	Facilities using a paper-based information system		Facilities using Simple application^a^		
	Median	IQR	Median	IQR	
Direct hypertension activities	110	71–195	101	71–124	.22
Indirect hypertension activities	39	26–62	15	11–19	<.001
Non-noncommunicable disease activities	60	46–87	110	63–139	.02

a Open source software (10).

b Mann-Whitney *U* test for comparing medians.

## Discussion

In this time and motion study, nurses spent nearly two-thirds of their time on hypertension activities in the primary care facilities of 2 Indian states. Nurses working in clinics with paper-based systems spent more time on documentation and record management than did those in clinics using the Simple app. Time and motion studies have helped managers to understand workforce efficiency in various healthcare settings, such as outpatient departments, intensive care units, and operating theaters (17–19). This time and motion study has helped managers understand the workforce efficiency of specific clinical, documentation, and management tasks.

In our study, nurses spent 52% of their time on direct and 14% on indirect hypertension activities. Similar research assessed the activities and time spent by the community health officers (CHOs, nurses with additional training in India), at health and wellness centers, which manage to up to 5,000 patients. The CHOs spent 40% of their time on NCD service delivery, primarily on hypertension and diabetes-related activities, which is consistent with our study’s results (20).

In our study, nurses spent approximately 66% of their total time on hypertension activities. In the primary health centers, a staff nurse does hypertension work and other duties such as outpatient care. Because of challenges in recruitment, training, and re-allocating tasks, nurses are either not appointed or are unable to do exclusive NCD-related work (21,22). Evidence suggests that multitasking might influence the nurses’ quality of care (23). Primary care facilities currently manage less than a quarter of the estimated population with hypertension (24). Even with the current coverage, nurses have to spend a large proportion of time in hypertension activities. We need to use this information to design more efficient hypertension services in the future. Apart from ensuring the appointment of nurses and protecting their time in the forenoon for hypertension services, other strategies such as the involvement of other health care workers also need to be explored.

Our study documented the multiple tasks performed by the staff nurses on a particular day in the primary care settings. Nurses spent 34 minutes on blood pressure measurement, 35 minutes on its documentation, and 23 minutes on patient counseling per day. Nurses spend less time on patient education and health promotion, an essential aspect of chronic disease care, than on other activities. (25). Sharing tasks between the available employees of the primary care setting may help create more efficient allocation of activities, especially during the busy hours before midday. Evidence from low-income and middle-income countries in the context of diseases such as HIV/AIDS and tuberculosis documented that task sharing is viable for addressing human resource constraints and providing a cost-effective approach (26,27). Paramedical staff or volunteers can help in measuring blood pressure and documentation in NCD clinics. Community health volunteers in NCD clinics have the potential to improve screening services and care of patients (28). The pharmacist can dispense and explain the medication schedules to the patient. As the NCD program expands, patients with hypertension and other comorbidities will require more complex care, which will be time-consuming and resource intensive. In addition, as caseloads increase, differentiated service delivery models, including clinical visits for stable patients every 3 or 6 months, will be needed to improve health care system functioning and convenience for patients.

Clinics using a paper-based system spent almost twice the time in recording patient data and clinic management compared with clinics using the Simple app. The Simple app is an efficient electronic health record management tool, designed in collaboration with health care workers, requiring minimal time to register and record a follow-up visit. Simple enables a health care worker to register a patient in 45 seconds and to record a follow-up visit in 15 seconds (10). The digital system eliminates paper-based cards, hence the need for organizing and retrieving cards for follow-up. Reports are automatically generated through the app dashboard, reducing the burden of manually compiling data and preparing reports. Evidence from a meta-analysis of 11 studies that investigated the impact of digitalization on nurses’ efficiency documented that using electronic health records could save 24.5% of time (29). Another novel intervention to reduce blood pressure measurement time is installing arm-in blood pressure monitors in the NCD clinics. The Government of Thailand documented using automated arm-in digital blood pressure measurement devices with an attached printer in their NCD clinics as a best practice (30). The system can use staff with less training than nurses to help the patients measure blood pressure in a validated arm-in blood pressure monitor.

Our study has several limitations. First, 2 research assistants observed the activities of the staff nurses in the NCD clinics. We gave detailed training to avoid interobserver bias and used standardized data collection tools and similar operational definitions for each activity. Staff nurses can change their behavior if they know they are observed (31). We minimized this Hawthorne effect by observing the staff nurses for 7 days, so that the nurses didn’t know which observations were being recorded. Second, the ease of managing the NCD clinic and the Simple app depended on the staff nurses’ experience and training. Although all 6 nurses in our study had completed the training, they had varying skills in managing the clinic. Third, we conducted the study in 6 facilities in 2 states. The health system functioning and status of implementation of NCD activities may vary from state to state. Still, study findings may be robust because the diagnosis, treatment, and recording policies and formats are the same in the 2 states. Fourth, our study included 6 primary care facilities from districts participating in the IHCI program. The time nurses spend on hypertension activities might be higher in our sites compared with facilities in the non-IHCI districts. IHCI strengthens the hypertension control activities in a primary care facility through standardized treatment protocols, follow-up guidelines, and intense training of nurses in managing the clinic and data management (14). IHCI also conducts supervisory and monitoring visits to these centers to ensure the quality of care delivered to individuals with hypertension (14). Hence, the findings are not generalizable to districts where IHCI is not being implemented.

Our findings suggest that nurses’ substantial time commitment and practical digital tools can improve the functioning of the staff nurse in India’s primary care settings. The digital Simple app system reduced the time spent on nonclinical indirect hypertension-related activities. Introducing user-friendly digital tools that require minimal time for data entry and that provide analytical dashboards can increase the time spent on patient-centric hypertension control activities. We recommend designating or appointing a staff nurse for hypertension-associated activities in the outpatient settings in primary care facilities. The health system will need to adopt time-saving methods such as task sharing and arm-in blood pressure apparatus to improve the efficiency of hypertension care.
